# Training needs of health researchers in research ethics in Cameroon: a cross-sectional study

**DOI:** 10.1186/s12909-022-03767-z

**Published:** 2022-09-29

**Authors:** Jerome Ateudjieu, Ketina Hirma Tchio-Nighie, Fernando Kemta Lekpa, Ingrid Marcelle Koutio Douanla, Frank Forex Kiadjieu Dieumo, Paul Nyibio Ntsekendio, Felicité Naah, Cavin Epie Bekolo, Anne Cecile Bisseck

**Affiliations:** 1 Department of Health Research, M.A. SANTE (Meilleur Accès aux Soins de Santé), P.O. Box: 3390, Yaounde, Cameroon; 2 Division of Health Operations Research, Cameroon Ministry of Public Health, Yaounde, Cameroon; 3 Department of Public Health, Faculty of Medicine and Pharmaceutical Sciences, University of Dschang, Dschang, Cameroon; 4 Internal Medicine Department, Douala General Hospital, Douala, Cameroon; 5 Department of Internal Medicine, Faculty of Medicine and Pharmaceutical Sciences, University of Dschang, Dschang, Cameroon; 6 National Agency for AIDS Research, Yaounde, Cameroon

**Keywords:** Research ethics, Training needs, Cameroon, Health researchers, Africa, Research participants’ protection, Ethical evaluation

## Abstract

**Background:**

Researchers are responsible for the protection of health research participants. The purpose of this study was to identify and prioritize the training needs of researchers involved in human health research in Cameroon.

**Methods:**

It was a cross-sectional study conducted in all the Cameroon regions in the last quarter of 2020. It targeted researchers involved in human health research selected by systematic stratified sampling from health and training institutions, and health facilities. Data were collected using a face-to-face administered questionnaire deployed in Smartphones via the ODK-collect. The distribution of participants’ exposure to research ethics training was described as well as their knowledge on the related regulatory texts. A score was used to rank the training needs identified by the participants.

**Results:**

Of 168 reached participants, 134 (79.76%) participated in the study. A total of 103 (76.87%) researchers reported having received training in human health research ethics and 98 (73.13%) perceived need of training in research ethics. Of those involved in clinical, vaccine, and field trials, 63.64, 33.33, 52.53% have been exposed respectively to related training regarding participants’ protection. Having received at least one training in research ethics significantly increase the proportion of researchers systematically submitting application for ethical evaluation prior to implementation (OR = 3.20 (1.31–7.78)). Training priorities identified by researchers include: guidelines and regulations on health research ethics and research participant’s protection in Cameroon, procedures for evaluating research protocols, protection of research participants in clinical trials, and fundamental ethics principles.

**Conclusion:**

The coverage of researchers in training regarding research participant protection remains limited in a number of areas including those related to clinical trial participant protection and research participant protection in Cameroon. Improving this coverage and addressing perceived needs of researchers are expected to contribute in improving their ability in playing their role in research participant protection.

## Introduction

Health Research is an indispensable tool in understanding health phenomena and ensuring population access to healthcare [1]. This requires most of the time interaction of research teams with human subjects [1]. These interactions in some cases expose research participants to serious risks and trauma [2–4]. Despite the availability of regulation, ethical misconduct or questionable research practices is still being reported [2].

A set of ethical guidelines has been put in place at the international level to promote and standardize the regulation of research participants protection in countries and to guide researchers [5–9]. To appropriately understand and use these guidelines during the planning and implementation of research project, researchers who are key actors with major responsibility in protecting research participants require a minimum training in specific domains [10].

Trainings offered to improve research participant’s protection are expected to be oriented according to trainee’s perceived and objectively needs, as underlined in previous studies assessing training needs in research ethics in human health among medical students and members of research ethics committees in sub-Saharan Africa [10–12]. A study conducted in Cameroon reported limited access of medical students to training in research ethics and to existing national and international regulation in health research participants’ protection [11]. This is supposed to limit their ability to protect research participants during their theses and during research projects that some of them will implement in their future as researchers.

Exploring researchers training needs in research participants’ protection is expected to guide decision-makers and researchers in responding to these needs, thus contributing to improving the protection of research participants and access to health research. The present survey was conducted with as aim to identify and prioritize the training needs of human health researchers for the protection of research participants in Cameroon.

## Method

### Study design

A cross-sectional study was conducted in all the Cameroon regions targeting researchers involved in human health research working in health training institutions, research institutes, health facilities, and ethics committees of Cameroon. Data were collected by trained and supervised surveyors using a face to face semi structured questionnaire.

### Study area and period

The study was carried out from November to December 2020. It covered all the 10 regions of Cameroon where health training institutions, research institutions, health facilities known to conduct research activities and research ethics committee of Cameroon.

### Participants

We considered a researcher to be any person involved as a principal investigator or co-investigator in the planning and/or implementation of a research protocol implemented in Cameroon. All researchers who had been a principal investigator or co-investigator on a research project in selected institutions were included. Students implementing their thesis and researchers who refused to participate were excluded.

### Sampling

All health and training facilities known to be involved in health research in each Cameroon regions were targeted. In each of the selected institutions, one third of the researchers were contacted for interview. They were selected through systematic stratified sampling. Strata were made of basic training categories in which potential participants were randomly selected.

### Data collection tool

Data were collected using a face-to-face questionnaire administered by trained surveyors to researchers in different institutions via a digital questionnaire implemented on the Kobo Toolbox and deployed on the tablets through the Open Data Kit (ODK) app. The questionnaire were adapted from previous studies [11]. The adapted version was pretested on 8 researchers based in Yaounde Cameroon. Main variables of the questionnaire included main domain of research in which researchers have been involved, previous exposure to training, access and use of local and international regulations, attitude regarding application for ethical clearance and main perceived training needs of researchers on research ethics.

### Data collection procedures

Heads of selected institutions were contacted and presented aims, targets and procedure of the survey. Those who consented to involve their institution were asked to provide the list of researchers of their institution. Potential participants were selected from these lists, contacted, informed about the study and invited to participate. Those consenting to participate were administered a face-to-face questionnaire. Those who could not be met after three visits were considered unreached, and those refusing to participate were considered as non-respondents.

### Data quality control

Data cleaning was ensured at two levels. At field level, all collected data were reviewed by a supervisor for improvement regarding completeness and reduction of outliers before transmission to the online data base. The data base was downloaded at daily base and processed by qualified data managers in order to identify and correct outliers and inconsistencies, and to ensure completeness with the participation of field teams where needed.

### Data analysis

We estimated with a 95% confidence interval the proportions of: participants previously exposed to training in research ethics distributed per type of training, participants identifying given training needs, participants’ access and use of local and international regulations, participants’ attitude regarding application for ethical clearance.

To determine priority training needs, participants were asked to identify and rank their first 4 training priorities. Points were assigned to the attributed rank in descending order, with the highest points representing the first rank. Thus, the first priority received 4 points, the second 3 points, the third 2 point and the fourth 1 point. The mean of points attributed to each training content was estimated with 95% confidence interval. Training contents were ranked in descending order and the first 5 were selected as the training priorities.

### Ethical consideration

This study was conducted to identify and prioritize the training needs of human health researchers for the protection of research participants in Cameroon. All participants were informed of the objectives and procedures of the survey and only those who consented to participate were included and interviewed. The validation of the informed consent was by a signature on a consent form. No data judged to be confidential was collected from respondents. Data were coded and was accessible only by the data manager staff and investigators’ team. All methods related to the study were performed in accordance with the Declaration of Helsinki. The research protocol of the study was approved by the Cameroon National Ethics Committee for Human Health Research (N^o^2020/10/1305/CE/CNERSH/SP).

## Results

### Characteristics of human health researchers

**Fig. 1:**
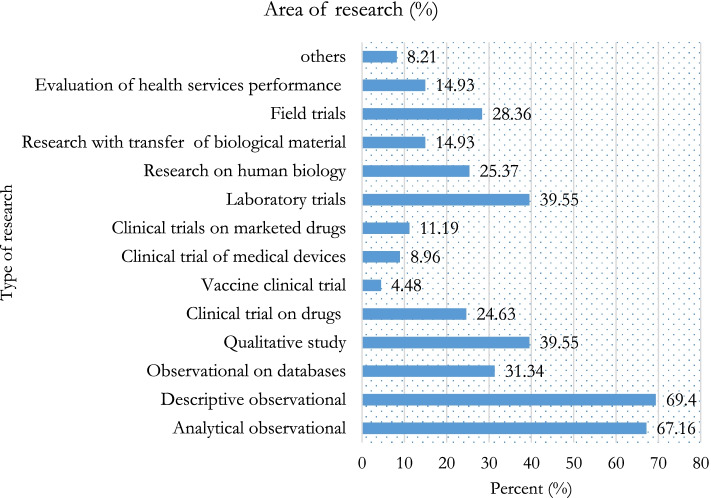
Area of research of human health researchers

Out of the 167 human health researchers reached, 134 (80.24%) agreed to participate in the survey. Table 1 gives the distribution of participants per region and the response rate in each of these regions. The centre region was the most represented with 74 (44.31%) reached participants. No researcher was reached in the South region during the data collection period. Table 2 presents the distribution of participants per institution, sex and basic trainings. The most represented professional category was medicine with 44 participants (32.84%). Forty (29.85%) participants were female. The most represented type of institution was health training institutions with 65 participants (48.51%). Figure 1 presents the distribution of research domains in which participants claimed to have been involved. Most of the researchers (93(69.40%)) had previously been exposed to descriptive observational studies and a small proportion exposed to vaccine clinical trials (6(4.48%)).

**Table 1:** Distribution of reached and consented health human researchers per region

Region	Reached per region		Consented in each region	
	n	Proportion (%)	n	Proportion (%)
Adamawa	4	2.40	3	75.00
Centre	74	44.31	54	72.97
East	5	2.99	5	100.00
Far North	2	1.20	2	100.00
Littoral	24	14.37	19	79.17
North	10	5.99	7	70.00
North West	4	2.40	4	100.00
West	16	9.58	15	93.75
South West	28	16.77	25	89.29
South	0	00.00	0	00.00
**TOTAL**	**167**	**100.00**	**134**	80.24

**Table 2:** Main characteristics of human health researchers

Characteristics	***N*** = 134	
	n	%
**Institutions of human health researchers**^**a**^		
Health Facilities	29	21.64
Researches institutes	40	29.85
Health training institutions	65	48.51
**Sex**		
Female	40	29.85
Male	94	70.15
**Respondent’s basic training**		
Biology	41	30.60
Medicine	44	32.84
Pharmacy	7	5.22
Public Health	15	11.19
Social Sciences	6	4.48
Statistics	1	0.75
Others	20	14.93

^a^Some participants had more than one institution

### Previous exposure to training

A total of 103 (76.85%) researchers reported having received training in human health research ethics. Domains covered by these training are presented in Table 3. It is noted that the coverage of exposure to training varies per domain. Table 4 indicates among those previously involved in trials design studies, those who have been exposed to training on research participant’s protection during clinical trials including vaccine trials, and good clinical practices. It is noted that 46.27, 30.59 and 32.83% of participants previously involved in clinical, vaccine and field trials have not been exposed to training regarding the protection of participants involved respectively in those study types.

**Table 3:** Exposure of participants to training in research ethics

Themes on training received	(***N*** = 134)	
	n	%
**Fundamental principles of health research ethics**	72	53.73
**International texts regulating research ethics**	70	52.23
**Role of the investigator in protecting research participants**	64	47.76
**Good Clinical Practices**	63	47.01
**Protection of research participants in clinical trials**	62	46.27
**Procedures for submitting research protocols to ethics committee**	57	42.54
**National regulations for research participants’ protection in Cameroon**	54	40.30
**Procedures for evaluating research protocols**	52	38.81
**Protection of research participants with transfer of biological materials**	51	38.06
**Evaluation of the protection of vulnerable people during the implementation of the research protocol**	51	38.06
**Procedures for monitoring the implementation of research protocols**	50	37.31
**Protection of participants in field trials**	44	32.83
**Protection of research participants during vaccine trials**	41	30.59
**Organization of research in Cameroon**	39	29.10
**Other**	8	5.97

**Table 4:** Coverage of related training among participant previously involved in trials studies

Type of trials to which some participants have been involved	Frequency of participants claiming to have been involved: n/134(%)	Coverage of training on research participants’ protection in related trial: n (%)	Coverage of training in good clinical practices: n (%)
Clinical trials of drugs	33(24.63)	21(63.64)	23(69.70)
Vaccine trials	6(24.63)	2(33.33)	3(50.00)
Field trials	38(24.63)	20 (52.53)	26(68.42)

### Source of previous training to which participants have been exposed

**Fig. 2:**
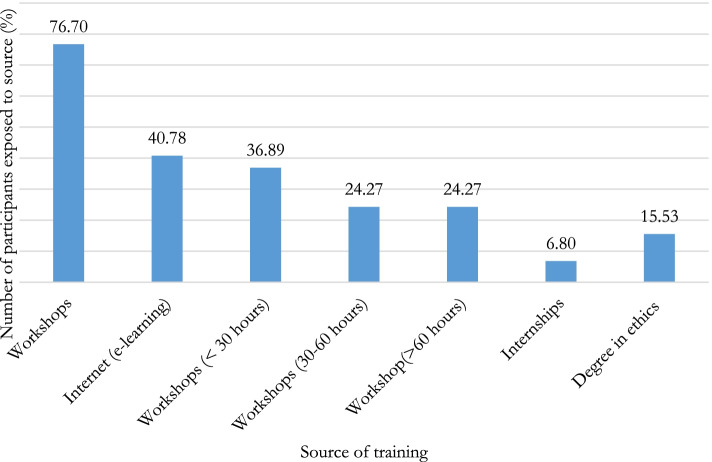
Source of previous training to which participants have been exposed

Figure 2 presents source of previous training to which participants have been exposed. It is noted that the most frequent source to which participants claim to have been exposed are workshops (79(76.70%)) and online training (42 (40.78%)).

### Previous exposure to regulation in health research ethics

Table 5 shows respondents’ prior exposure to research participants’ protection regulations. Access to and use of these texts was heterogeneous but relatively low for local texts.

**Table 5:** Exposure to regulations in research participant’s protection

Guidelines	Known		Read		Used	
Regulatory tests	n	%	n	%	n	%
**Declaration of Helsinki**	89	66.42	82	61.19	69	51.49
**Nuremberg Code**	70	52.24	57	42.54	42	31.34
**Belmont Report**	39	29.10	30	22.39	24	17.91
**CIOMS guideline**	34	25.37	29	21.64	24	17.91
**ICH/Good Clinical Practice**	70	52.24	57	42.54	42	31.34
**Order establishing Research Ethics Boards**	39	29.10	30	22.39	24	17.91
**Decision on obtaining Administrative Research Authorizations (ARA)**	34	25.37	29	21.64	24	17.91

### Submission of application to ethical clearance for previous studies

Out of the 134 surveyed researchers, 105 (78.37%) declared to always request an ethical evaluation prior to their research implementation, 20 (14.93%) declared to sometimes request and 9 (6.72%) declared to never request. Having received at least one training in research ethics significantly increase the proportion of researchers systematically submitting application for ethical evaluation (OR = 3.20 (1.31–7.78)).

Of the 39 researchers involved in clinical trials of either drugs, vaccines or medical devices, 35 (89.74%) declared to always apply for ethical clearance prior to their research implementation. Table 6 presents the distribution of application to ethical clearance per main research focus of participants.

**Table 6:** Distribution of application to ethical clearance per main study focus designs of participants

Study design	Frequency		Apply for ethical evaluation	
	n	%	n	%
Analytical observational	90	67.16	72	80.00
Descriptive observational	93	69.40	73	78.49
Observational on databases	42	31.34	32	76.19
Qualitative study	53	39.55	42	79.25
Clinical trial on drugs	33	24.63	29	87.88
Clinical trial on vaccines	6	4.48	4	66.67
Clinical trial of medical devices	12	8.96	11	91.67
Clinical trials on Marketed Drugs	15	11.19	13	86.67
Laboratory Trials	53	39.55	42	79.25
Research on human biology	34	25.37	24	70.59
Research with transfer of biological material	20	14.93	17	85.00
Field Trial	38	28.36	33	86.84
Health services performance evaluation	20	14.93	17	85.00
Others	11	8.21	9	81.82

### Perceived training needs in research ethics

Of the 134 human health researchers surveyed, 98 (73.13%) perceived need of training in research ethics. Identified training needs are presented in Table 7. The training areas with the most perceived needs were fundamental principles of health research ethics (72 (53.73%)), international regulations for research participants’ protection (70 (52.73%)), role of the investigators in the protection of participants (64 (47.76%)); and good clinical practises (63 (47.01%)).

**Table 7:** Participants’ identified training needs

Training area	(***N*** = 134)	
	n	%
**Fundamental principles of health research ethics**	72	53.73
**International regulations for research participants’ protection**	70	52.24
**Role of the investigator in protecting participants**	64	47.76
**Good Clinical Practices**	63	47.01
**Role of researchers in the protection of research participants in clinical trials**	62	46.27
**Procedures for submitting application for ethical clearance**	57	42.54
**National regulations for research participants’ protection in Cameroon**	54	40.30
**Procedures for evaluating application for ethical clearance**	52	38.81
**National and international regulation regarding the transfer of biological samples and data sharing**	51	38.06
**Role of researchers in the protection of vulnerable people during the implementation of the research protocol**	51	38.06
**Procedures for monitoring of ethical issues during the implementation of research protocols with ethical clearance**	50	37.31
**Role of researchers in the protection of participants during field trials**	44	32.84
**Role of researchers in the protection of research participants during vaccine trials**	41	30.60
**Organization of ethical evaluation in Cameroon**	39	29.10
**Others**	8	5.97

### Perceived training priorities

The ranking of scores attributed by participants to the perceived priority training topics are presented in Table 8. This ranking showed that the four priority subjects on which the researchers would like to be trained are: (1) Guidelines and regulations on health research ethics and research participant’s protection in Cameroon, (2) Procedures for evaluating research protocols, (3) Protection of research participants in clinical trials, and (4) Fundamental ethics principles.

**Table 8:** Researchers training needs priorities

Ranking	Sum of Scores	Training needs
1	87	Guidelines and regulations on health research ethics and research participants protection in Cameroon
2	63	Procedures for evaluating research protocols
3	59	Protection of research participants in clinical trials
4	40	Fundamental ethical principles
5	28	Procedures for monitoring the implementation of research protocols
6	18	Drafting ethical consideration in a research protocol
7	15	Protection of research participants in field trials
8	13	Good Clinical Practices
9	11	Role of the investigator in protecting research participants
10	7	Planning and implementing Material Transfer Agreement on health research
11	7	Functioning of ethics committees
12	3	Obtaining informed consent
13	3	Bioethics and health research
14	3	Conducting Risk benefit ratio assessment
15	3	Community involvement in health research

### Preferred training sources perceived by researchers

**Fig. 3:**
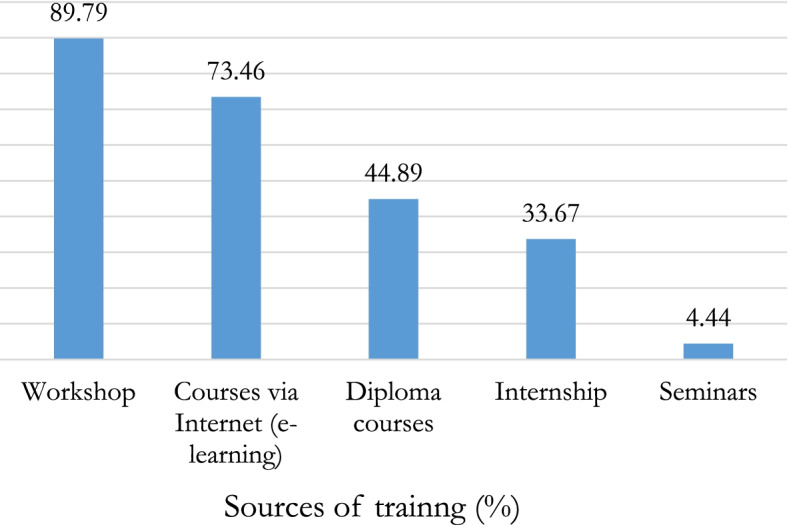
Sources of training most preferred

Figure 3 presents the training sources preferred by researchers for their training. Workshops (89.79%) and e-learning (73.46%) were the main source of training desired by researchers who expressed the need to be trained. Internships and seminars were the least preferred source of training.

## Discussion

This study reveals that 76.85% of researchers has been exposed to training in research ethics with workshops and online training as most frequent sources of training. Of those involved in clinical, vaccine, and field trials, 63.64, 33.33, 52.53% have been exposed respectively to related training regarding participants’ protection. Having received at least one training in research ethics significantly increase the proportion of researchers systematically submitting application for ethical evaluation prior to implementation (OR = 3.20 (1.31–7.78)). Training priorities identified by researchers include: guidelines and regulations on health research ethics and research participant’s protection in Cameroon, procedures for evaluating research protocols, protection of research participants in clinical trials, and fundamental ethics principles.

The Helsinki Declaration attributes the responsibility of ensuring the protection of research participants to researchers and the duty to be trained in research ethics before being involved as investigator in a given research protocol [5]. Given that there is actually no data available that describes these prerequisites among Cameroonian researchers, the present study was necessary to provide needs of intervention in terms of training of researchers on research participants’ protection. The study reveals that around one out of four researchers involved as investigator in research protocols declare to have not been exposed to any training in research ethics. Despite the importance of this indicator in monitoring the level of countries in protecting research participants, very few studies have addressed it. A study conducted earlier in Nigeria documented a more serious situation with up to 36% of researchers participating in protocol implementation with no training in research ethics [13]. Our findings support the importance of training as tool for capacities improvement of researchers as it revealed that being trained increases the proportion of researchers always applying for ethical evaluation prior to their protocol implementation. The consistency of training contributing in capacity building is supported in other domains [14, 15]. Given that the protection of research participants involves the mastery of a given number of ethical considerations, it is necessary to ensure that the training received by researchers covers these considerations [16]. The present study indicates that the coverage of researchers exposed to each training domain varied. This underlines that having received a training does not imply the ability to adequately respond to different ethical issues that may be part of a given research protocol. It underlines the need to standardize in any context the training curricula of researchers.

Clinical trials are traditionally known to expose to higher risks than other study designs. In line with this, specific international guidelines have been developed to address ethical issues when implementing this type of studies [6, 9]. Mastery of the implementation of related ethical considerations should be taken into account in the capacity building of any researcher planning to be involved in clinical trials [17]. The present study reveals that whatever the trial design, at least 30% of researchers involved in clinical, vaccine or field trials were not exposed to any related training before the protocol implementation. No data is available in main publication databases to assess the consistency of these findings in other contexts. The fact that up to 30% of researchers have been involved in clinical trials with no exposure to training questions their ability to detect and adequately respond to ethical issues during the protocol implementation. This finding underlines the need of implementing the national law regulating medical research that states in its article 32 that the training of researchers is mandatory prior to medical research implementation [18].

Taking into account the perception of the beneficiaries of a public health intervention is expected to contribute to the success of this intervention [19, 20]. About three quarters of participants reported the need for training in research ethics. The training topics identified as needs by participants were diverse with the most frequent being Fundamental principles of health research ethics and international regulations for research participants’ protection. Participants also identified their training priorities with the top priority topics being guidelines and regulations on health research ethics and research participant’s protection in Cameroon, procedures for evaluating research protocols, protection of research participants in clinical trials, and fundamental ethics principles. Similar needs and priorities were identified by members of ethics committees in three African countries [10]. The combination of perceived training needs and priorities cover at least 5 key domains of training that may capacitate researchers in applicable regulation and ethical principles, in understanding the monitoring and evaluation of their research projects or in responding to ethical issues during clinical trials. To the best of our knowledge, this is the first time the training of researchers has been documented in the Cameroon context. Given that the results cover key competencies as recommended in guidelines, it can be used as starting point to plan the training of researchers.

The source and method of health training determine the effect of the training on the trainees’ abilities [2]. The majority of participants of the present study opted for workshops, followed by online training as preferred source of training. To the best of our knowledge, no study documents significant difference between exposure to any training source and its outcome on trainees’ competencies. Given the necessity of increasing ethical standards of studies conducted in developing countries and the scarcity of training resources and sources to prepare researchers, we would recommend to researcher to be trained via the most accessible source presented to them.

The results of the present study must be considered with certain limitations. The process of recruitment of participants only targeted institutions and did not cover independent researchers. Despite the fact there is no information estimating the proportion of independent of researchers in Cameroon, we assume that their proportion in Cameroon is limited. Up to 20% of reached researchers did not consent to participate and no information was collected on the reasons. We cannot predict what would have been the effect of their response on the proportion of participants exposed to training and training needs. Despite these limitations, we believe that the study covered a large proportion of researchers and the majority of research areas and the results generated can be used to plan the response to needs and training priorities of researchers.

## Conclusion

About three quarters of Cameroonian researchers have been exposed to training in research ethics even if the distribution of coverage per training topics varied. The proportion of researchers exposed to training in good clinical practices and ethical issues regarding drug clinical trials, vaccine clinical trials and field trials was relatively low among researchers involved in these study designs. About four out of five researchers claim to always apply for ethical evaluation prior to protocol implementation and being exposed to training significantly increases the proportion of researchers applying for ethical evaluation. Despite the fact a relatively great proportion of researchers had already been exposed to training, they expressed the need for training in a wide range of topics and identified as priority topics: guidelines and regulations on health research ethics and research participant’s protection in Cameroon, procedures for evaluating research protocols, protection of research participants in clinical trials, and fundamental ethics principles.

We recommend to each researcher involved in health research to make sure to acquire a minimum training applicable to the field of research in which he is involved before the implementation of these protocols. We also recommend to authorities involved in the regulation of research ethics to develop a standardized training program covering each research field and to include the evaluation of this training in the ethical review process. Finally, we propose to actors involved in capacity building of researchers in the field of research participant protection to take into account the priorities identified by them, that include guidelines and regulations on health research ethics and research participant’s protection in Cameroon, procedures for evaluating research protocols, protection of research participants in clinical trials, and fundamental ethics principles.

## Data Availability

The datasets used and/or analyzed during the current study are available on: 10.13140/RG.2.2.18158.48966.
